# Microscopic colitis in alopecia areata: A gut-skin connection beyond the follicle

**DOI:** 10.1016/j.jdin.2025.08.003

**Published:** 2025-08-25

**Authors:** Milan Hirpara, Rachel Greene, Makenna Chapman, Carl Gregory Albers, Natasha Mesinkovska

**Affiliations:** a Department of Dermatology, University of California, Irvine, Irvine, California; b Division of Gastroenterology, Department of Medicine, University of California, Irvine Medical Center, Orange, California

**Keywords:** alopecia areata, celiac disease, collagenous colitis, Crohn’s disease, eosinophilic esophagitis, lymphocytic colitis, microscopic colitis, ulcerative colitis

*To the Editor:* While alopecia areata (AA) is an autoimmune disease of the hair follicle, its systemic associations remain underappreciated. At our institution, we observed an unexpected pattern of young AA patients with a history of abdominal pain, bloating, and diarrhea since childhood without a clear diagnosis. In several of these cases after extensive workup, the final answer was surprising: microscopic colitis (MC), a T-cell mediated gastrointestinal inflammatory disease rarely seen in young adults. Genome wide studies support AA association with gastrointestinal conditions, such as celiac and inflammatory bowel disease (IBD).^1^ To further explore this colitis relationship, we conducted a retrospective cohort study using a global research database (TriNetX), focusing on AA and immune-mediated gastrointestinal conditions, with an emphasis on MC.

We generated a cohort of patients with AA and matched them by age, sex, and race to healthy controls. ICD-10 codes were used to identify the prevalence of immune-mediated gastrointestinal conditions. Age-stratified analysis was conducted for MC. Odds ratios (OR) were calculated by TriNetX and validated by a statistician.

After propensity score matching, 117,674 patients with AA were compared to matched controls (mean age: 33.5 ± 20.7 years; 60.9% female) (Table I). Patients with AA had significantly higher odds of MC (OR = 1.88; *P* < .001), including both lymphocytic (OR = 1.83; *P* < .001) and collagenous colitis (OR = 1.80; *P* = .003). Among those with MC, patients with concurrent AA were younger (59.6 vs 62.8 years; *P* = .003) and more often female (79% vs 74%) (Table II). Age-stratified analysis revealed that the risk of MC was elevated in both the 41-64 year (OR = 1.59) and 65+ year (OR = 1.59) age groups. Other immune-mediated gastrointestinal diseases were also significantly more common in AA: celiac disease (OR = 1.87), Crohn disease (OR = 1.75), eosinophilic esophagitis (OR = 1.59), and ulcerative colitis (OR = 1.38).

**Table I:** Patient demographic information for alopecia areata and control before and after propensity score matching for age, sex, and race (top). Risk of developing immune-mediated gastrointestinal conditions and age-stratified outcomes for microscopic colitis in alopecia areata cohort compared to control cohort after propensity score matching (bottom)

Characteristics	Before matching		After matching		
	AA	Control	AA	Control	SMD
Overall, n	117,674	12,020,328	117,674	117,674	-
Age at index, mean (SD)	33.5 ± 20.7	48.7 ± 19.9	33.5 ± 20.7	33.5 ± 20.7	<0.001
Sex, n (%)					
Female	71,639 (60.9)	6,546,108 (54.5)	71,639 (60.9)	71,641 (60.9)	<0.001
Male	43,588 (37.0)	4,928,968 (41.0)	43,588 (37.0)	43,588 (37.0)	<0.001
Unknown	2447 (2.1)	545,252 (4.5)	2447 (2.1)	2447 (2.1)	<0.001
Race, n (%)					
White	57,350 (48.7)	7,201,054 (59.9)	57,350 (48.7)	57,350 (48.7)	<0.001
Black or African American	17,041 (14.5)	1,381,742 (11.5)	17,041 (14.5)	17,043 (14.5)	<0.001
Asian	8657 (7.35)	481,869 (4.01)	8657 (7.35)	8657 (7.35)	<0.001
Other Race	8178 (6.95)	500,077 (4.16)	8178 (6.95)	8176 (6.95)	<0.001

Gastrointestinal immune-mediated condition	AA	Control	OR	95% CI	*P*-value
Overall, n	117,674	117,674	-	-	-
Other noninfective colitis, n (%)	2819 (2.40)	960 (0.82)	**2.98**	**2.77-3.21**	**<.001**
Microscopic colitis, n (%)	216 (0.184)	115 (0.098)	**1.88**	**1.50-2.36**	**<.001**
0-40 y∗	19 (0.032)	15 (0.025)	1.27	0.64-2.49	.686
41-64 y∗	65 (0.176)	41 (0.111)	**1.59**	**1.07-2.35**	**.020**
65 + y∗	116 (0.661)	80 (0.456)	**1.45**	**1.09-1.93**	**.010**
Lymphocytic colitis, n (%)	106 (0.090)	58 (0.049)	**1.83**	**1.33-2.52**	**<.001**
Collagenous colitis, n (%)	72 (0.061)	40 (0.034)	**1.80**	**1.22-2.65**	**.003**
Celiac Disease, n (%)	600 (0.510)	322 (0.274)	**1.87**	**1.63-2.14**	**<.001**
Crohn’s disease, n (%)	730 (0.620)	418 (0.355)	**1.75**	**1.55-1.98**	**<.001**
Eosinophilic esophagitis, n (%)	396 (0.337)	249 (0.212)	**1.59**	**1.36-1.87**	**<.001**
Ulcerative colitis, n (%)	756 (0.642)	550 (0.467)	**1.38**	**1.23-1.54**	**<.001**

-Indeterminate and eosinophilic colitis were omitted due to lack of significance and low prevalence, respectively.

-*P*-values were adjusted for multiple comparisons using the Benjamini-Hochberg false discovery rate correction.

-International Classification of Diseases, 10th Revision (ICD-10) codes for diagnoses are as follow: AA = L63; other noninfective colitis = K52.8; microscopic colitis = K52.83; lymphocytic colitis = K52.832; collagenous colitis = K52.831; celiac disease = K90.0; Crohn disease = K50; eosinophilic esophagitis = K20.0; ulcerative colitis = K51.9.

-The bold-italic values represent the average percent discount. These values were derived using the following formula: Average % Discount = (Gross Sales−Net Sales)/Gross Sales.

*AA*, Alopecia areata; *CI*, confidence interval; *n*, number of patients; *OR*, odds ratio; *RD*, risk difference; *SD*, standard deviation; *SMD*, standardized mean difference.

∗ Values represent cumulative prevalence, and each age group is not mutually exclusive.

**Table II:** Demographic characteristics of patients with microscopic colitis with and without alopecia areata

Characteristics	MC and AA	MC without AA	*P*-value
Overall (n)	254	63,119	
Age at index, mean (SD)	59.6 ± 15.6	62.8 ± 16.2	**.003**
Sex, n (%)			
Female	200 (79)	46,719 (74)	.110
Male	39 (15)	12,954 (21)	.064
Race, n (%)			
White	213 (84)	53,871 (85)	.483
Black/African American	10∗ (4)	1367 (2)	.074†
Asian	10∗ (4)	415 (1)	**<.001**†
American Indian/Alaska Native	10∗ (4)	152 (1)	**<.001**†
Native Hawaiian or Pacific Islander	10∗ (4)	530 (1)	**<.001**†
Other Race	10∗ (4)	1158 (2)	**.0286**†
Ethnicity, n (%)			
Hispanic or Latino	10∗ (4)	1523 (2)	.127†
Not Hispanic or Latino	198 (78)	45,256 (72)	.0532

-*P*-values were adjusted for multiple comparisons using the Benjamini-Hochberg false discovery rate correction.

-International Classification of Diseases, 10th Revision (ICD-10) codes for diagnoses are as follow: AA = L63; MC = K52.83.

*AA*, Alopecia areata; *MC*, microscopic colitis.

∗ The value is between 1 and 10, listed as 10 for patient privacy.

† These *P*-values may not be statistically reliable, as TriNetX rounds event counts fewer than 10 up to 10.

The immune dysregulation and microbial disruption in AA may contribute to the associations observed in this study. Prior literature links AA to celiac disease and IBD through shared polymorphisms in CTLA4, IL-2/21, MICA, and certain human leukocyte antigens (HLA).^1^ Although no direct genetic link has been established with MC, both AA and MC involve Th1/Th17-mediated cytokine activation, a hallmark of many autoimmune diseases.^1^^,^^2^ Further, both conditions have shown responsiveness to Janus kinase inhibitors, which downregulate Th1 and Th17 signaling pathways.^3^ Collagenous colitis exhibits a more pronounced Th1 response and an HLA-association, suggesting a stronger link to AA.^2^^,^^4^ In our cohort, both subtypes had similarly elevated ORs in AA patients. Gut microbiota disruption may also contribute to MC pathogenesis in AA patients as both conditions have decreased microbial diversity and increased pro-inflammatory bacterial species.^5^

We noted an earlier age of MC onset in AA patients; however, we were unable to determine the implications of MC in pediatric AA cases due to privacy-based data restrictions (<10 cases). Pediatric underdiagnosis of MC may be associated with lower colonoscopy rates.

Additional limitations include the retrospective design and potential ICD-10 code misclassification. This study reinforces the association between AA and gastrointestinal immune-mediated conditions, underscoring the importance of screening in the AA population and gastroenterology referral when patients present with chronic digestive symptoms.

## Conflicts of interest

None disclosed.
